# Premarital sex and condom use among trainee healthcare workers: an exploratory study of selected healthcare training institutions in Enugu State, Nigeria

**DOI:** 10.11604/pamj.2019.32.7.14749

**Published:** 2019-01-04

**Authors:** Obinna Ositadimma Oleribe, Obehi Hilda Okojie, Nicholas Jonathan Burstow, Simon David Taylor-Robinson

**Affiliations:** 1 Excellence and Friends Management Care Centre (EFMC), Dutse Abuja FCT, Nigeria; 2 Department of Community Medicine, University of Benin, Benin City, Edo State, Nigeria; 3 Liver Unit, Department of Surgery and Cancer, St Mary’s Hospital Campus, Imperial College London, Praed Street, London, W2 1NY, United Kingdom

**Keywords:** Premarital sex, healthcare workers, Enugu, Nigeria

## Abstract

**Introduction:**

To assess the prevalence and causes of premarital sex and condom use among trainee healthcare workers in selected healthcare institutions in Enugu State, Nigeria; and to proffer solution to challenges identified.

**Methods:**

We used a mixed study approach with qualitative and quantitative components. Informed consent was obtained from participants and data collected using self-administered structured questionnaires. Epi info® was used for data analysis.

**Results:**

A total of 362 respondents (309 unmarried) from four healthcare training institutions participated in the study. Among unmarried respondents, 141 (45.8%) were sexually active. Premarital sex was more common among Pentecostals and sexual activity increased with age (r=0.78; p <0.05). Premarital sexual activity was more common among males and trainee nurses (p <0.005). Although knowledge of condom use was high, actual use was poor (20.1%), with lowest rates among females, Catholics and age-group 30-35 years. Breakages, high failure rates and reduced sexual satisfaction were cited as major factors responsible for poor use. Use of non-specific terms such as "casual sex" and "casual or regular sex partners" hindered consistent, correct condom use.

**Conclusion:**

There is a significant gap between knowledge of and actual use of condoms, despite high premarital sexual activity amongst healthcare workers. Furthermore, non-specific terminologies hinders appropriate condom usage. We propose the term: Committed Spousal Partner (CSP) defined as "a sexual partner who commits to fidelity (one sexual partner per time) and whose current HIV status is known through medical testing and is properly documented" in place of all non-specific terminology.

## Introduction

Human immune deficiency virus (HIV) infection with resultant acquired immune deficiency syndrome (AIDS) is one of the few diseases almost entirely preventable through simple measures. Despite this, close to 40 years after the discovery of HIV in the 1980s, the disease is still spreading. Sexual intercourse (including men having sex with men) has remained a major source of transmission of HIV across the world [1], as has unprotected premarital sex and sex with sex workers. Peer pressure and the broadcast media also contribute, instigating adolescents into risky behavior and illicit substance use, while facilitating the spread of disease [2]. Premarital sex among students is a common phenomenon [3-6] and is of particular importance because adolescent and young adults between 15-24 years of age account for a vast proportion of newly acquired sexually transmitted diseases [7]. Another facilitator of the spread of sexually transmitted diseases is the improper, sporadic, or lack of use of condoms, which may be due to a number of factors, including: pressure from male partners not to use protection, alcohol consumption prior to sexual intercourse increasing risk-taking behaviour, need for money forcing women into becoming sex workers and rape [8]. In a study of sexually active locals from Java, Indonesia, 60% did not take any action to prevent sexually-transmitted diseases (STD) or pregnancy during their last sexual encounter [9]. Other studies have shown that condom use varies with geographical location [10] and is low with regular partners, even amongst high-risk populations like injecting drug users [9]. Healthcare workers should be knowledgeable of HIV and have a favorable attitude towards its prevention [11]. Although there have been studies on premarital sex and condom usage among students [3-6], none has specifically focused on students in medical and allied healthcare professions. The objective of this study was to assess the prevalence of premarital sex and the use of condoms among trainee healthcare workers in selected healthcare institutions in Enugu State, southeastern Nigeria. It was also designed to document their level(s) and appropriateness of condom use. To achieve these objectives, three research questions were used: (1) What is the prevalence of premarital sex among students in selected institutions of higher learning?; (2) What is the prevalence of condom use during premarital sex among students in the selected institutions of higher learning?; (3) How effective is the current condom use campaign in Nigeria?

## Methods

A mixed study approach in which qualitative content was imbedded in a quantitative study technique using a cross-sectional study design was conducted in selected healthcare training institutions in Enugu, Nigeria. Participants were trainees in these selected healthcare training institutions. The study population comprised undergraduates in schools of: basic nursing; post-graduate nursing; midwifery; and the Institute of Medical Laboratory Science, all affiliated to the University of Nigeria Teaching Hospital, Enugu. A total sample size of 362 against the minimal sample size of 264 calculated using standard technique was used. A cluster sampling technique was used to enlist participants into the study, surveying all students from randomly selected classes. For the School of Postgraduate Nursing, which only had one class, all students in the class at the time of survey were included in the study. Data collection was carried out over a period of four weeks, with the last group of students sampled on World AIDS day (December 1^st^ 2016). Research permission was obtained from the University of Benin. We also received consent from the participating institutions and participants before their inclusion in the survey. A pre-tested Self-Administered Questionnaire (SAQ) was used for data collection. Epi info® (https://www.cdc.gov/epiinfo) and MS Excel were used for data analysis.

**Ethical approval**: This article is a cross sectional study and does not contain any studies with human participants or animals performed by any of the authors.

## Results

**Figure 1:**
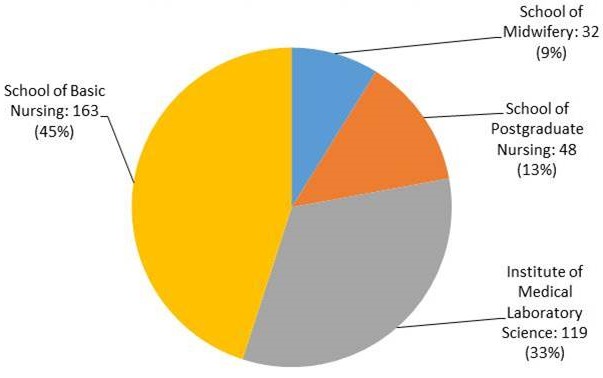
study participants by discipline

A total of 362 respondents from the four healthcare training institutions were enlisted in the study. Of these 362, 361 respondents returned their self-administered questionnaires, giving a response rate of 99.7%. However, some questionnaires were not completed fully (Table 1). The majority of participants were drawn from the School of Nursing (Figure 1). Of the 361 participants, 308 (85.1%) were unmarried. Close to 80% of all participants were females (247, 79.9%). The mean age of participants was 24.41 ± 5.6 years, and the majority identified as Catholic (173, 58.6%). Radio was the main source of reproductive and sexual health information.

**Table 1:** Demographic characteristics of participants

Age Group	Frequency	Percentage (%)
15 - 19	39	12.7
20 - 24	189	61.4
25 - 29	65	21.1
30 - 34	13	4.2
35 - 39	2	0.6
> 40	0	0.0
Total	308	100.0
**Religion**	**Frequency**	**Percentage (%)**
Catholic	173	58.6
Anglican	68	23.1
Pentecostal	41	13.9
Muslim	1	0.3
Other denominations	12	4.1
Total	295	100.0
**Sources of Information**	**Frequency**	**Percentage (%)**
Radio	245	79.29
Television	206	66.67
Teachers	216	69.90
Health workers	254	82.20
Friends	194	62.78
Others (e.g. siblings’ parents’ internet, books seminars etc.)	47	15.21

**Knowledge of HIV/AIDS and condoms:** A total of 298 participants (96.4%) knew that AIDS is an infection, while 277 (89.6%) knew that HIV is a virus. The highest level of knowledge was found among postgraduate nurses and midwives (100%), while levels of knowledge were lower among students of the Institute of Medical Laboratory Science (94.6%) and first year basic nursing students (94.6%). Wrong responses given included: *"AIDS is a curse", "HIV is an infection" and "HIV means Higher Immune Vaccine".* The level of knowledge between the different participating schools were not significantly different at 95% CI (X^2^ = 0.52; p>0.05) All participants had heard of condoms, but only 290 (93.8%) had previously seen one. While all midwifes and post-graduate nursing students had previously seen condoms, rates of 80.3% were reported in first year nursing students. Generally, more of the sexually active participants had seen a condom before, but this difference was not statistically significant (p>0.05).

**Attitude to HIV/AIDS and condoms:** One hundred and ten (35.6%) respondents believed that they were at risk of HIV. More of the sexually active participants accepted they were at risk of HIV infection than the non-sexually active (36.6% vs. 23.4%, respectively) (X = 7.44, P<0.01). Fifty-eight (18.8%) respondents reported that they would have sex (with or without condoms) if they were HIV positive. This was reported as being in order to satisfy their sexual needs, as sex was seen as a basic human right that needed to be satisfied by 22 (57.9%) of respondents. Thirty-three (56.9%) respondents reported that they would have sex if HIV was status positive, either to satisfy their desires or that of their partner(s) or spouse. Other reasons are listed in Table 2.

**Table 2:** Reasons why people desire to have sex and the impacts of the condom campaign on HIV prevention

Reasons why people with HIV still desire to conceive	Frequency	Percentage (%)
Right to procreation	25	34.7
God’s commandment to multiply	9	12.5
To maintain family lineage	11	15.3
Fulfill womanhood	27	37.5
Total	72	100.0
**Common positive impacts of the Condom Campaign on HIV prevention and control**		
Increased awareness of the disease	34	34.7
Increased condoms availability	31	31.6
Increased condom use	23	23.5
Increased peoples’ fear of the disease	10	10.2
Total	98	100.0
**Common negative impacts of the Condom Campaign on HIV prevention and control**		
Increased sexual laxity	38	36.9
Increased moral decadence	27	26.2
Increased cheap sex	26	25.2
Carefree attitude about sex/prostitution	12	11.7
Total	103	100.0

To support her desire to have sex with or without a condom, one respondent said: *"There is nothing else to prevent transmission".* Another said: *"I am still a human being"*. Of all those who would have sex with a positive HIV status, 42 (72.4%) reported that they would use a condom for every sexual encounter; and 10% reported that they would have sex only with HIV positive individuals. Sexually active respondents were significantly more likely to report that they would have sex with HIV positive partner(s) than those who were not. ((56, 28.9% vs. 11, 6.6%, respectively). X^2^ = 29.46, P<0.005). Two hundred and one (65%) respondents believed that condom awareness campaigns and condom usage have affected the spread of HIV, with 98 (48.8%) claiming that the effects have been positive (Table 2). Concerning condoms, some participants said: *"it made fornication and adultery become rampant", "it has enhanced the spread of HIV and AIDS by making people carefree about sex", "it makes our boys and girls engage in casual sex", "Many people go into prostitution because there is condom availability".*

**Figure 2:**
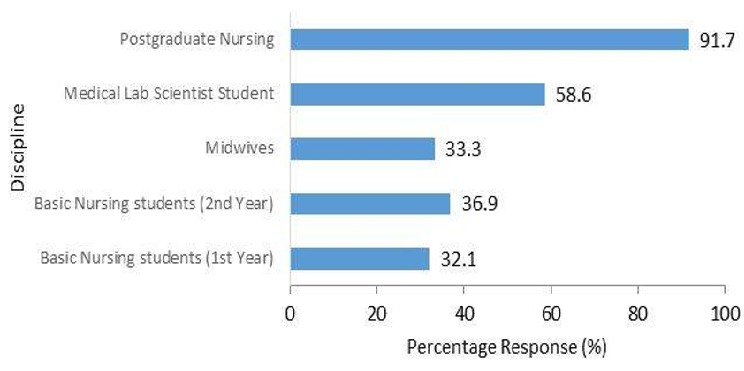
Proportion of respondents who were sexually active by discipline

**Sexual and condom use, practice and behavior:** Of the unmarried respondents, 141 (45.8%) were sexually active. Males were significantly more likely to be sexually active than females (68.8% vs. 40.4%, respectively, X^2^=13.18, p<0.005). The highest proportion of sexual activity in unmarried respondents was seen in postgraduate nursing students (91.7%) (Figure 2). Interschool differences in sexual activity were not statistically significant, except between trainee nurses and medical laboratory science students (X^2^=14.4, P<0.005). Increased age was associated with increased levels of sexual activity (r=0.78, t=2.16, P<0.05) (Table 3). Premarital sex was more common among Pentecostal Christian adherents, but this difference was not statistically significant. Over 80% (113) of sexually active respondents had ever used a condom, with the lowest usage rates in first year basic and postgraduate nursing students. All had heard of condoms and over 93% had seen a condom. More males than females used condoms (84.8% vs. 81.0%, respectively) but this difference was not significant. Of those who had ever used condoms, 86 (76.1%) used a condom during their last sexual activity, and 39 (34.5%) use a condom during every sexual encounter. Sixty-one percent and 27.7% of sexually active singles used condoms in their last encounter and during every sexual activity, respectively. Institutional analyses revealed that only 22% of the Enugu Institute of Medical Laboratory School students and 27% of postgraduate nursing students used condoms during every sexual encounter.

**Table 3:** Proportion of respondents who were sexually active and use condoms by age and religious group

Sexually active by age		
Age (Years)	Frequency	Proportion
15-19	9	0.23
20-24	82	0.43
25-29	39	0.6
30-34	9	0.69
35-39	2	1
Total	141	
**Sexually active by religion**		
**Religious group**	**Frequency**	**Proportion**
Catholic	76	0.44
Anglican	28	0.41
Pentecostal	20	0.49
Muslim	1	1
Others	7	0.58
Total	132	100
**Use condom by age**		
**Age (Years)**	**Frequency**	**Proportion**
15-19	6	0.67
20-24	70	0.85
25-29	30	0.77
30-34	5	0.56
35-39	2	1
Total	113	1
**Use condom by religion**		
**Religious groups**	**Frequency**	**Proportion**
Catholic	58	0.76
Anglican	24	0.86
Pentecostals	16	0.8
Others	7	0.88
Total	105	100

**Reasons for sexual activities, effectiveness of condom and ease of use:** The majority of participants had sex to satisfy personal desires (238, 77%). Most used condoms to prevent infections (218, 59.9%), but cited "tearing" as a major cause of condom ineffectiveness (80, 25.9%) and the belief that one's partner was safe was cited as a hindrance to the use of condoms (Table 4). Participants had several complaints against the use of condoms including: that they were *easy to forget when aroused (143), troublesome to use (119), had reduced sensation (93), opposition from sexual partners (87), general unavailability (60), breakage and busting (55), interruption of pleasure (49), embarrassment to buy* (44) or that their *usage brought with it suspicion of respondent infidelity* (36). Other reasons cited included condoms being: *uncomfortable* (27), *irritating (22), brought with them a requirement for being careful (16), that condoms often come off during sex (16), that usage might make a partner think the respondent had AIDS (15), ineffectiveness (11), that they slip (11), that they spoilt the intimacy of the sexual act* (9), that they were *embarrassing to discard* (8), or that the respondent *did not like how a condom felt* (6). Several of the respondents had more than one complaint. Eighty-four (27.3%) respondents believed that condoms were effective in protecting individuals against infections, 137 (44.5%) and 116 (37.7%) against sexually transmitted infection and HIV/AIDS (Table 5). Although a higher proportion of sexually active respondents agreed that condoms were effective, condoms were said to have failed some of the respondents either by breaking, slipping off, or the female partner getting pregnant in 31 cases (27.4%), among those that had ever used condoms. Breakage was the most frequent cause of failure, accounting for 67.7% of all failures.

**Table 4:** Reasons why people have sex, use condoms and hinders of condom use

Why people have sex		
Reasons	Frequency	Percentage (%)
To satisfy personal desires	238	77.0
To satisfy partners	184	59.5
To sustain relationship	152	49.2
To enhance finances	121	39.2
To make children	110	35.6
To satisfy curiosity	17	5.5
No reason	37	12.0
**Why people use Condoms**		
**Reasons**	**Frequency**	**Percentage**
To prevent pregnancy	185	59.9
To prevent infection	218	70.6
To prevent HIV/AIDS	150	48.5
Others (e.g. To feel safe)	5	1.6
**Why Condoms are ineffective**		
**Problems**	**Frequency**	**Percentage**
Tear	80	25.9
Irregular use	76	24.6
Inconsistent use	69	22.3
Poor quality	58	18.8
Holes in condoms	47	15.2
**Beliefs hindering use of condoms**		
**Beliefs**	**Frequency**	**Percentage**
Meant for prostitutes	44	14.2
Do not need one	115	37.2
Cannot get infected	69	22.3
Knew that their partner(s) were safe or uninfected	198	64.1
Protect self by other means	34	11.0
Not at risk of HIV infection	19	6.1

**Table 5:** Effectiveness of condoms against HIV infection, pregnancy and other infections

Sexual activity				
Effectiveness of condoms against	Yes	No	X^2^	p-Value
HIV/AIDS	84	32	22.03	<0.005
Pregnancy	137	77	22.35	<0.005
Infections (other STIS)	116	57	23.69	<0.005

## Discussion

There was a significant difference between levels of premarital sexual activity of trainee nurses, when compared with medical laboratory trainees. Male trainees were more sexually active than females. Despite high premarital sexual activity amongst surveyed healthcare workers, condom use was poor. Although knowledge of condom use was high, actual and consistent use was poor, resulting in a gap between knowledge of condom usage and actual practice. This finding is similar to studies from across the world. For instance, in an Indian study, nearly half of respondents (48.4%) used condoms inconsistently even amongst female sex workers and those engaging in anal sex with other men [12]. A study in Singapore reported consistent condom use with paid or casual partners of 39.6% and 36.2% for vaginal and oral sex, respectively [13]. Another study designed to examine prevalence and determinants of condom use among female undergraduates at 16 university campuses in China revealed 18.1% having sexual intercourse, with 19.8% having used a condom in their first sexual encounter [5]. In that study, 30% of those having intercourse reported never, seldom or sometimes using condoms in the past 12 months [5]. A Canadian study using a national sample of 653 Canadian university students reported higher condom usage amongst men than women (55.4% and 42.3%, respectively) [14], similar to the findings of this study. Being over 25 years of age, not a manual laborer and the perception that the respondent might be at risk of HIV, were factors that positively affected condom usage [12, 13]. Also in the Canadian study, female students who had sex with a more committed partner had slightly lower odds of reporting condom use at last penile vaginal intercourse [14]. A Philippine study revealed that 42% of the study population did not always use condoms [15], assertions echoed in other studies [16]. These findings, similar to the findings of this study, show that although premarital sex rates are high, condom use is poor, even among healthcare students. Poor condom use is worsened by the use of non-specific terms like "casual sex" and "casual or regular-sex partners". As these words are poorly defined, there is a need for a more specific terminology that explicitly specifies what is needed for safer sex among unmarried sexually active people.

## Conclusion

We found substantial gaps between knowledge, attitude and practice, revealing considerable unmet needs for family live education. This significant gap exists between the knowledge of and actual use of condoms, despite high premarital-sexual activity amongst surveyed trainees. Use of non-specific terms such as casual-sex, casual and regular-sex partners hindered appropriate use of condoms. This should be replaced. We propose the use of Committed Spousal Partner (CSP) in place of all non-specific and poorly defined terms. We define a CSP as: "a sexual partner who commits to fidelity (one sexual partner per time) and whose current HIV status is known through medical testing and is properly documented". A committed spousal partner's status can change to non-committed spousal partner when any of these conditions are violated. To achieve CSP, there is the need to support sexually active unmarried people to know their HIV status, offer counseling to couples and advocate for improved and consistent use of condoms where HIV status is unknown (i.e. when the partner is not a CSP). Furthermore, there is the need to actively program to close the gap between knowledge and practice among trainee healthcare workers. Anthropological studies should be commission to identify sociological factors that may influence knowledge and practice.

### What is known about this topic

- Premarital sex is on the increase across the world including within healthcare training institutions;
- Comprehensive sex and reproductive life education prevents unwanted pregnancies, infections including HIV and AIDS, and other sex related health challenges;
- With the advent of HIV/AIDS, use of condoms with casual sex partners is one of the core preventive messages along with abstinence and faithfulness to one's sex partner in the ABC of Prevention.

### What this study adds

- Poor condom use is seen even among healthcare workers, including those in medical school and health training institutions;
- "Casual sex partner" is an ill-defined term. We propose the use of Committed Spousal Partner (CSP) in place of all non-specific and poorly defined terms. We define a CSP as: "a sexual partner who commits to fidelity (one sexual partner per time) and whose current HIV status is known through medical testing and is properly documented." A committed spousal partner's status can change to non-committed spousal partner when any of these conditions are violated.

## Competing interests

The authors declare no competing interests.

## References

[1] Mayer KH, Wang L, Koblin B (2014). Concomitant socioeconomic, behavioral and biological factors associated with the disproportionate HIV infection burden among Black men who have sex with men in 6 US cities. PloS one.

[2] Kamath VG, Kamath A, Roy K, Rao CR, Hegde A, Ashok L (2016). A qualitative study on how adolescent males in South India view reproductive health. International Journal of Adolescent Medicine and Health.

[3] Ma Q, Ono-Kihara M, Cong L (2008). Unintended pregnancy and its risk factors among university students in eastern China. Contraception.

[4] Regassa T, Chala D, Adeba E (2016). Premarital Sex in the last twelve months and its predictors among students of Wollega University, Ethiopia. Ethiopian Journal of Health Sciences.

[5] Tang L, Chen R, Huang D (2013). Prevalence of condom use and associated factors among Chinese female undergraduate students in Wuhan, China. AIDS Care.

[6] Zhang L, Gao X, Dong Z, Tan Y, Wu Z (2002). Premarital sexual activities among students in a university in Beijing, China. Sexually Transmitted Diseases.

[7] Nikula M, Gissler M, Jormanainen V, Sevon T, Hemminki E (2009). Sexual behaviour and lifestyles of young men in Finland, 1998-2005: cross-sectional survey of military conscripts. The European Journal of Contraception & Reproductive Health Care.

[8] Dada JO, Olaseha IO, Ajuwon AJ (1997). Sexual behavior and knowledge of AIDS among female trade apprentices in a yoruba town in South-Western Nigeria. International Quarterly of Community Health Education.

[9] Saktiawati AMI, Worth H, Lazuardi E, Spooner C, Subronto YW, Padmawati RS (2013). I Just trust him: the notion of consideration as a barrier to condom use amongst women who inject drugs in Central Java. World Journal of AIDS.

[10] Rasamimari A, Dancy B, Talashek M, Park CG (2007). Predictors of sexual behaviors among Thai young adults. The Journal of the Association of Nurses in AIDS Care: JANAC.

[11] Hedayati-Moghaddam MR, Moradi Marjaneh M, Mashhadi IE (2012). Knowledge and attitudes of physicians in private practice towards HIV/AIDS in Mashhad, Iran. International Journal of STD & AIDS.

[12] Ramanathan S, Ramakrishnan L, Goswami P (2013). O23.2 Correlates of inconsistent condom use during anal sex with female sex workers (FSWs) among male clients: survey findings from three high prevalence States of India. Sexually Transmitted Infections.

[13] Lim RBT, Wong ML, Cheung ON, Tham DKT, Tai BC, Chan R (2017). Factors associated with consistent condom use and STIs among foreign female entertainment workers: results from a cross-sectional survey in Singapore. Sexually Transmitted Infectionsl.

[14] Milhausen RR, McKay A, Graham CA, Crosby RA, Yarber WL, Sanders SA (2013). Prevalence and predictors of condom use in a national sample of Canadian university students. The Canadian Journal of Human Sexuality.

[15] Urada LA, Morisky DE, Hernandez LI, Strathdee SA (2013). Social and structural factors associated with consistent condom use among female entertainment workers trading sex in the Philippines. AIDS and Behavior.

[16] Shaw SY, Bhattacharjee P, Isac S (2013). A cross-sectional study of sexually transmitted pathogen prevalence and condom use with commercial and noncommercial sex partners among clients of female sex workers in southern India. Sexually transmitted diseases.

